# Discovering cryptic species in the Aspiciliella intermutans complex (Megasporaceae, Ascomycota) – First results using gene concatenation and coalescent-based species tree approaches

**DOI:** 10.1371/journal.pone.0216675

**Published:** 2019-05-28

**Authors:** Zakieh Zakeri, Volker Otte, Harrie Sipman, Jiří Malíček, Paloma Cubas, Víctor J. Rico, Veronika Lenzová, David Svoboda, Pradeep K. Divakar

**Affiliations:** 1 Senckenberg Museum of Natural History, Görlitz, Germany; 2 Botanischer Garten & Botanisches Museum Berlin-Dahlem, Berlin, Germany; 3 The Czech Academy of Sciences, Institute of Botany, Průhonice, Czech Republic; 4 Departamento de Farmacología, Farmacognosia y Botánica (U.D. Botánica), Facultad de Farmacia, Universidad Complutense, Madrid, Spain; 5 Charles University in Prague, Faculty of Sciences, Department of Botany, Prague, Czech Republic; National Cheng Kung University, TAIWAN

## Abstract

Taxonomic identifications in some groups of lichen-forming fungi have been challenge largely due to the scarcity of taxonomically relevant features and limitations of morphological and chemical characters traditionally used to distinguish closely related taxa. Delineating species boundaries in closely related species or species complexes often requires a range of multisource data sets and comprehensive analytical methods. Here we aim to examine species boundaries in a group of saxicolous lichen forming fungi, the *Aspiciliella intermutans* complex (*Megasporaceae*), widespread mainly in the Mediterranean. We gathered DNA sequences of the nuclear ribosomal internal transcribed spacer (nuITS), the nuclear large subunit (nuLSU), the mitochondrial small subunit (mtSSU) ribosomal DNA, and the DNA replication licensing factor MCM7 from 80 samples mostly from Iran, Caucasia, Greece and eastern Europe. We used a combination of phylogenetic strategies and a variety of empirical, sequence-based species delimitation approaches to infer species boundaries in this group. The latter included: the automatic barcode gap discovery (ABGD), the multispecies coalescent approach *BEAST and Bayesian Phylogenetics and Phylogeography (BPP) program. Different species delimitation scenarios were compared using Bayes factors species delimitation analysis. Furthermore, morphological, chemical, ecological and geographical features of the sampled specimens were examined. Our study uncovered cryptic species diversity in *A*. *intermutans* and showed that morphology-based taxonomy may be unreliable, underestimating species diversity in this group of lichens. We identified a total of six species-level lineages in the *A*. *intermutans* complex using inferences from multiple empirical operational criteria. We found little corroboration between morphological and ecological features with our proposed candidate species, while secondary metabolite data do not corroborate tree topology. The present study on the *A*. *intermutans* species-complex indicates that the genus *Aspiciliella*, as currently circumscribed, is more diverse in Eurasia than previously expected.

## Introduction

The lichen-forming fungal family *Megasporaceae* Lumbsch, Feige & K. Schmitz is a monophyletic group, belonging to the Ascomycota [1]. Its representatives are characterized by their mostly crustose thallus, urceolate or lecanorine apothecia, world-wide distribution and predominantly saxicolous, muscicolous, but also terricolous and lignicolous habit [1–6]. Some members of *Megasporaceae* (e.g. *Aspicilia cinerea* (L.) Körb., *Circinaria caesiocinerea* (Nyl. ex Malbr.) A. Nordin, Savić & Tibell, known *C*. *calcarea* (L.) A. Nordin, Savić & Tibell, *C*. *contorta* (Hoffm.) A. Nordin, Savić & Tibell, *Lobothallia radiosa* (Hoffm.) Hafellner) are widely distributed and very common throughout Europe, and they significantly contribute to the saxicolous communities on various types of rocks. Nevertheless, the taxonomic concept of many species remains unclear and many lichenologists consider this family dauntingly difficult.

The currently accepted taxonomy of *Megasporaceae* includes seven genera: *Aspicilia* A. Massal., *Circinaria* Link, *Lobothallia* (Clauzade & Cl. Roux) Hafellner, *Megaspora* (Clauzade & Cl. Roux) Hafellner & V. Wirth, *Sagedia* Ach., *Teuvoa* Sohrabi & S. Leavitt, and *Aspiciliella* M. Choisy in Werner [7–8]. The genus *Aspiciliella* [9] was resurrected only recently based on three phylogenetic markers: ITS, mtSSU and nuLSU [5]. The study confirmed that *Aspiciliella intermutans* (Nyl.) M. Choisy in Werner and *A*. *cupreoglauca* (B. de Lesd.) Zakeri, Divakar & Otte, previously assigned to *Aspicilia* based on their general appearance, represent a distinct lineage (*Aspiciliella*) in *Megasporaceae* and form a monophyletic clade together with the newly described species *A*. *portosantana* Sipman & Zakeri [5]. Species of the genus are characterized by a rimose-areolate, partially continuous, K+ red thallus and a chlorococcoid photobiont. The apothecia are pale brown to dark grey or black, they are rarely surrounded by a distinct thalline margin and a proper exciple is always present, the epihymenium is green to olive-green to greenish-brown, N+ changing to light green. The exciple is hyaline. Hypothecium and subhymenium are colorless, I+ blue to rusty red. The hymenium is colourless, I+ blue to rusty red, the asci are 8-spored, of *Aspicilia*-type. The ascospores are ellipsoid, colourless, simple. The conidia are straight, 7–11μm long [5,8]. *Lecanora intermutans* Nyl. was described from St. Laon (France) in 1872 [10]. Nylander [10] indicated in the original description that the species looks like *Aspicilia cinerea* but has larger ascospores 23–34 × 9–15 μm and smaller conidia 7–9 x 1 μm. Later, this species was transferred to the genus *Aspicilia* [11] and more recently to *Aspiciliella* [9,5]. *Aspiciliella intermutans* is widely distributed, especially in the Mediterranean Region. Collections are more abundant from Iran, Armenia, France and Greece. It is also very common in Italy [12] and the Iberian Peninsula [13]. In Central Europe, *A*. *intermutans* is rare and locally distributed. Its overall distribution reaches Great Britain [14], the Canary Islands [15], Tunisia (e.g. [16]), Syria [17], the Ural Mountains [18] and the U.S.A. [19], although not all of these occurrences were verified by DNA methods.

Our previous study has shown that *Aspiciliella intermutans* includes some well-supported clades that may represent a complex of unrecognized species [5]. This stimulated us to work more on this group to find out the phylogenetic relationships and to explore the possibility of cryptic speciation in this complex. Previous studies have shown the presence of cryptic species in numerous species complexes of mainly foliose and fruticose lichens (e.g. [20–25]). Recently, a growing number of methods for analyzing DNA sequence data in a coalescent-based framework is capable to critically evaluate species diversity in fungi [26]. The coalescent-based species delimitation methods have otherwise not been used until now in *Megasporaceae* species, particularly not in studies of the *A*. *intermutans* complex. We used a combination of phylogenetic strategies to delimit species in the *Aspiciliella intermutans* complex applying coalescent-based approaches and other recently developed DNA-based methods.

Estimating a species tree and species delimitation using coalescent methods for closely related taxa have proven very useful and have been used for lichenized fungi in some species complexes, e.g. in *Parmotrema reticulatum* (Taylor) M. Choisy, *Parmotrema pseudoreticulatum* (Tav.) Hale, *Rhizoplaca melanophthalma* (DC.) Leuckert and *Cladia aggregata* (Sw.) Nyl. [20–25].

The objective of this study is to delimit species boundaries within the *Aspiciliella intermutans* complex by implementing single- and multi-locus phylogenetic analyses, and coalescent-based species delimitation methods.

## Materials and methods

### Specimen sampling and phenotypic study

We gathered 90 samples from Asia and Europe; they were mostly collected by the authors in Iran, Armenia and the Czech Republic. Specimens were also obtained from the following herbaria: B, GZU, PRA, PRC, PL, CR and GLM.

Locations of sampels:

1. **Armenia,** Aragatsotn, Byurakan, Mts. Aragats, H20 road to lake Kari, after diversion to Amberd castle, alt. 2470 m, 40°24'54''N, 44°14'51''E, Z. Zakeri 16.06.2015 (GLM 40741).
2. **Armenia,** Aragatsotn, Byurakan, Mts. Aragats, H20 road, before diversion to Amberd castle, alt. 2200 m, 40°23'55''N, 44°15'07''E, Z. Zakeri 16.06.2015 (GLM 40763, 40562, 40745).
3. **Armenia,** Aragatsotn, Byurakan, Mts. Aragats, road connecting M3 to H20, alt. 1800 m, 40°24'48''N, 44°22'03''E, Z. Zakeri 21.06.2015 (GLM 40475, 40480, 40469, 40487, 40494).
4. **Armenia,** Ararat, Vedi, Urtsadzor, Khosrov Forest State Reserve, alt. 1390 m, 39°59’07”N, 44°53’51”E, Z. Zakeri 17.06.2015 (GLM 40503, 40499).
5. **Armenia,** Ararat, Vedi, Urtsadzor, Khosrov Forest State Reserve, alt. 1600 m, 40°00’42”N, 44°54’41”E, Z. Zakeri 17.06.2015 (GLM 40519).
6. **Armenia,** Shirak province, Artik, northern slope of Mt. Aragats, SE of Mets Mantash village, W of reservoir, alt. 2465 m, 40°33'17"N 44°6'34"E, basalt, S. Harutyunyan & H. Mayrhofer 2006 (GZU 280743).
7. **Armenia,** Vayots Dzor, Areni, Amaghu valley along Noravank Monastery road, alt. 1265 m, 39°41'54''N, 45°12'35''E, Z. Zakeri 23.06.2015 (GLM 39711).
8. **Armenia,** Vayots Dzor, Shatin, Yeghegis, alt. 1530 m, 39°52'03''N, 45°20'38''E, Z. Zakeri 23.06.2015 (GLM 39702).
9. **Armenia,** Vayots Dzor, Shatin, Yeghegis, alt. 1600 m,39°52'14''N, 45°23'26''E, Z. Zakeri 23.06.2015 (GLM 39729, 39727).
10. **Armenia,** Vayots´ Dzor province, area c. 13 km E of Vayk, near Artavan, c. 1.4 km SW of village, forest along rivulet, forest and forest edge, alt. 1940 m 39°39'6"N 45°36'20", E, M. Oganesian 2009 (GZU 28656).
11. **Azerbaijan,** in finibus Talysorum in regione montuosa zuvand vocata prope vicum Mistan in rupibus, alt. 1900 m, ca. 38°38'39.3"N, 48°26'07.7"E, V. Otte 2013 (GLM 38734, 38706).
12. **Bulgaria**, Eastern Rodopi Mts., Kadžali, Momčilgrad, Bregovo, in the valley of Varbica river ca 2 km E of the village, alt. 300 m, 41°28'N, 25°25'E, on rather acid volcanic rock, J. Vondrák 2004 (PRA/JV2151).
13. **Czech Republic**, Central Bohemia, Český kras, Černošice, Karlík: a diabase rocky-hill just N of the village, alt. 250 m, 49°56'17,4"N, 14°15'42,2"E, Z. Palice 2011 (PRA/ZP14789).
14. **Czech Republic**, Central Bohemia, Křivoklátsko, Králův Dvůr, Trubínský vrch Nature Monument, alt. 330–360 m, 49°56'39''N 13°59'46''E, J. Malíček 2010 & V. Lenzová 2013 (JM2640, PRC/VL25).
15. **Czech Republic**, Central Bohemia, Praha, PR Prokopské údolí: Albrechtův vrch Nature Monument, 50°12'56.4"N, 14°16'43.4"E, S exposed diabase rock, V. Lenzová 2015 (PRC/VL255).
16. **Czech Republic**, Central Bohemia, Praha, PR Prokopské údolí: Hemrovy skály, 50°2'34.6"N, 14°21'13.5"E, S exposed diabase rock, V. Lenzová 2015 (PRC/VL253).
17. **Czech Republic**, Central Bohemia, Praha, Řeporyje, 50°1'52.7''N, 14°18'53.3''E, diabase, V. Lenzová 2014 (PRM784504, PRC/VL254).
18. **France**, Bretagne, Loire-Atlantique, Frossay, chemin des Carris, sur rocher de gneiss, alt. 5m, 47°17'35.06"N, 1°52'23.06"W, J.Y. Monnat 2011 (CR 26741).
19. **France**, Languedoc-Roussilon, Pyrénées-Orientales, Nohédes, à proximité immédiate de la réserve naturelle de Nohédes, 170 m á ONO de Cortal, en bordure d´une lande à Cytisus purgans, près de la piste, sur petites parois de schiste non calcaire, alt. 1030 m, 42°37'49.44"N, 2°16'10.56"E, C. Roux 2009 (CR 25602).
20. **France,** Provence-Alpes-Côte d´Azur: Var, Évenos, tuj sub jak S de la kastelo de ÉVENOS, sur rokbloko el bazalto, alt. 350 m, 44°18'57.96"N, 6°37'53.04"E, M. Bertrand & C. Roux 2010 (UPS L-591195/CR 25790).
21. **Greece,** Cyclades Archipelago. Milos island, W part, Angathia Bay, alt. 0–360 m, ca. 24° 20.7' E, 36° 43.65' N, H. Sipman & Th. Raus 2012 (B 59827, 59969, 59825).
22. **Greece,** North Aegean Region, Lesbos: ca. 3 km E of Filia along road to Kalloni, alt. 2–700 m, 26° 09.55' E, 39° 15.29' N, H. Sipman & Th. Raus 2016 (B 62695, 61847, 61933, 62681, 62663, 62614, 62252, 61911, 62653, 62271, 62704, 62439, 62105).
23. **Iran,** Ardebil, Sarein, alt. 3044 m, around 38°12'21.2"N, 47°52'38.6"E, Z. Zakeri 06.2014 (GLM 40760, 49966).
24. **Iran,** Ardebil, Sarein, alt. 1898 m, ca. 38°8'35.0"N, 48°0'96"E, Z. Zakeri 06.2014 (GLM 49960, 49959, 49965, 49269, 49269).
25. **Iran,** Golestan, Azadshahr, alt. 1802 m, ca. 36°50'08"N, 55°21'39.19"E, Z. Zakeri 08.2014 (GLM 50003).
26. **Iran,** Khorasan Razavi, Quchan, alt. 1880–1992 m, ca. 36°54'53.1"N, 58°07'11.6"E, Z. Zakeri 2015 (GLM 49964, 49961, 49962, 49963).
27. **Iran,** West Azerbaijan, Urmia, alt. 1305 m, ca. 37°50'37.5"N, 45°34'47.5"E, Z. Zakeri 06.2014 (GLM 49270, 49271, 49967, 49267).
28. **Italy**, Sardinia: Monte Limbara Sud, Azienda Foresta Demaniale, an Abzweigung Richtung „Su Nodu Nieddu“, alt. 650 m, 40°46'N, 9°11'E, auf Granit, N. Nöske 1999 (B 368).
29. **Romania,** Munţii Măcin Mts; Brăila, Măcin, on top of hill with former quarry ca 1 km SW from Mt Cheia, 45°14'N, 28°08'E, granitoid rock, J. Šoun 2005 (hb. Šoun 82).
30. **Spain**, Prov. Zamora. SW of Zamora, towards Pereruala, Arroyo del Zape, alt. 630 m, 41°28.3'N, 5°47.2'W, conglomerate rock outcrops in steppe-like area, W-slope, H. Sipman 2000 (B 45379).
31. **Ukraine**, Mikolajivska oblast, Pervomaisk, pasture near village Liushniuvate, alt. 80 m, 48°10'15,30"N 30°26'55,17"E, on nutrient-rich granite boulder, J. Vondrák 2006 (PRA/JV5204).
32. **Ukraine,** Pervomaysk, Blagodatnoe, in valley of brook running to Yuzhniy Bug, on granite outcrop in steppe slope, J. Vondrák (PRA/JV7340).

Molecular data of specimens tentatively referred as *Aspiciliella intermutans* were analyzed using 66 ITS, 51 MCM7, 61 nuLSU and 73 mtSSU sequences as they have been shown to be useful to resolve phylogenetic relations in this group of lichenized fungi ([e.g. [1,5,6,27]). Five samples of *Aspiciliella cupreoglauca* were selected as outgroup, since this species is closely related to *Aspiciliella intermutans* [5].

Morphology and anatomy of the specimens were observed, using a Leica M165 C stereomicroscope, and a Leica DM 2500 P compound light microscope connected to a Leica MC 190 HD digital camera. Detailed observations of thallus anatomy, asci, ascospores and conidia were made on hand-cut sections of thallus, apothecia and pycnidia mounted in tap water.

Chemistry of the specimens was investigated by thin layer chromatography (TLC), using solvent systems B and C, and by spot tests, using KOH, C and I, applied directly on the lichen thalli [28].

### DNA extractions, PCR amplifications and sequencing

Total DNA was extracted from freshly collected material according to Park et al. [29] but with some modifications as described in Zakeri et al. [5,6].

The primer pair ITS1F [30] and ITS4 [31] was used for the PCR amplifications of the ITS region. For PCR amplification of the nuLSU region the primers LR0R, LR7 and LR5 [32], for the mtSSU region the primers mrSSU1 and mrSSU3R [33], and for the MCM7 region the primers Mcm7-1348rev [34] and X_mcm7_F [35] were used. PCR amplifications were performed in a 12.5 μL volume containing 2 μL undiluted DNA, 0.5 μL of each primer (10 mM), 6.4 μL of sterile water, 1 μL dNTP (2 mM), 1 μL s–buffer, 1 μL MgCl_2_, 0.1 μL Taq–polymerase (Peqlab). Primer details and PCR conditions are summarized in Table 1. Thermal cycling parameters were initial denaturation for 5 min at 95°C, followed by 30 cycles of 30 secs at 95°C, 30 secs at 54°C for amplifying ITS, 64°C for nuLSU, 59°C for mtSSU, 58°C for MCM7, and 1 min at 72°C, a final extension step of 3 min at 72°C was added, after which the samples were kept at 4°C. The amplification products were visualized by electrophoresis on 1% agarose gels and stained with ethidium bromide, and were purified by adding 2 μL ExoSAP (Exonuclease 1-shrimp alkaline phosphatase, USB) to 5 μL of the PCR products, followed by a heat treatment of 15 min at 37°C and 15 min at 80°C. Both DNA strands of the PCR product were sequenced on an ABI 3730 by the Bik-F Laboratory Center in Frankfurt am Main.

**Table 1:** Primers used for PCR amplification and sequencing of the nuclear ribosomal internal transcribed spacer 1, 5.8S and internal transcribed spacer 2 (ITS) region, the nuclear large subunit (nuLSU), the mitochondrial small subunit (mtSSU) ribosomal DNA and the DNA replication licensing factor MCM7.

Marker	Primer name	Forward primer sequence	Annealing Temperature (°C)	Reference
**ITS**	ITS1F:	5'-CTTGGTCATTTAGAGGAAGTAA-3'	54°C,	(Gardes & Bruns 1993)
	ITS4:	5'-TCCTCCGCTTATTGATATGC-3'	54°C,	(White et al. 1990)
**nuLSU**	LR0R	5'-ACC CGC TGA ACT TAA GC-3'	64°C	(Vilgalys & Hester 1990)
	LR7	5'- TAC TAC CAC CAA GAT CT-3'	64°C	(Vilgalys & Hester 1990)
	LR5	5'- ATCCTGAGGGAAACTTC-3'	64°C	(Vilgalys & Hester 1990)
**MCM7**	Mcm7-1348rev	5'-GAYTTDGCIACICCIGGRTCWCCC AT-3'	58°C	(Schmitt et al. 2009)
	X_mcm7_F	5'-CGT ACA CYT GTG ATC GAT GTG-3'	58°C	(Leavitt 2010)
**mtSSU**	mtSSU1	5'-AGCAGTGAGGAATATTGGTC-3'	59°C	(Zoller et al. 1999)
	mtSSU3R	5'-ATGTGGCACGTCTATAGCCC-3'	59°C	(Zoller et al. 1999)

### Sequence alignment

Sequence fragments generated for this study were assembled and edited using BioEdit v.7.0 (www.mbio.ncsu.edu/BioEdit/BioEdit.html). Sequence identity was confirmed using the mega-BLAST search function in GenBank (https://blast.ncbi.nlm.nih.gov/Blast.cgi). The sequences were aligned by the program MAFFT v.7 [36] on a web server (https://mafft.cbrc.jp/alignment/server/), implementing the E-INS-I alignment algorithm for nuLSU and G-INS-I alignment algorithm for all other markers, ‘200PAM/K = 2’ scoring matrix and with an offset value of 0.0, with the remaining parameters set to default values. Gblocks 0.91b (http://molevol.cmima.csic.es/castresana/Gblocks_server.html) was used to delimit and exclude ambiguously aligned positions, applying settings allowing for smaller final blocks, gap position within the final blocks and less strict flanking position [37].

### Phylogenetic analyses

Maximum likelihood (ML) analyses were performed for the single loci separately and for the concatenated dataset using the online version of IQ-TREE [38–40] with simultaneous inference of the optimal partitioning scheme and substitution models for each data partition. For concatenated dataset, a partition of seven subsets has been proposed (Group I intron, ITS1, 5.8S rDNA, ITS2, mtSSU, nuLSU and MCM7). Conflicts among the single gene trees were assessed using the tree topology and following the criterion of nodes bootstrap support equal or above 95% in one but below this value in the other tree for no conflict. No conflicts were found and thus all the datasets were concatenated. Branch support was estimated with the ultrafast bootstrap algorithm [41] based on 1000 bootstrap replicates and using a maximum of 1000 iterations and a minimum correlation coefficient of 0.99 as a stopping rule.

The ITS PCR product obtained was around 800 bp. The larger sizes of ITS were due to the presence of insertions of about 200 bp identified as Group I intron [42] at the 3’ end of the SSU rDNA. Group I intron were present in all the samples of *Aspiciliella intermutans* complex.

We used the Markov Chain Monte Carlo approach implemented in MrBayes v.3.2.2 [43] to infer phylogenetic trees applying the partitioning scheme inferred with IQTREE and slightly simplified substitution models, because most of the models inferred by IQ-TREE are not implemented in MrBayes. See Table 2 for details on locus partitions and substitution models. Two independent runs, each with four heated Metropolis–Coupled MCMC chains (“temperature” 0.2) were initiated and run for 5,000,000 generations, with tree and parameter sampling every 100^th^ generations. Convergence among the parameters of the both runs was assessed in Tracer v.1.6 following the criterion of effective sample size (ESS) values above 200. The consensus tree was generated after discarding the initial 25% trees.

**Table 2:** Details on locus partitions and substitution models.

	Group I intron	ITS1	5.8S rRNA	ITS2	nuLSU	MCM7	mtSSU
**No. taxa**	61	69	69	69	65	51	77
**Position**	1–183	184–414	415–553	554–725	726–1798	1799–2305	2306–3205
**Substitution model**	TIM2e+G4	TIM2e+G4	TVMe+I+G4	K2P+I+G4	TPM3+I+G4	TNe+I	F81+I+G4

### Species delimitation analyses

In order to infer the most likely species numbers in our *Aspiciliella intermutans* dataset, we chose a method for single-locus DNA-based species delimitation, the automatic barcode gap discovery [44], and two coalescent-based models *BEAST [45] and BPP v.3.2 [46] to analyze the four loci concatenated dataset.

Our exploratory analyses showed that the ITS region is very variable in the *A*. *intermutans* group and the nuLSU is very conserved (results not shown) and thus they were not used for the final single-locus species delimitation analyses.

ABGD is an automatic procedure that sorts the sequences into hypothetical species based on the barcode gap. This method automatically finds the distance where the barcode gap is located [44]. The ABGD method was carried out for the mtSSU and MCM7 datasets using the Web interface at http://wwwabi.snv.jussieu.fr/public/abgd/abgdweb.html. Default parameters were chosen using Kimura 2-parameter (K2P) distances that correct for transition rate bias (relative to transversions) in the substitution process. The default for the minimum relative gap width was set to different values between 0.1 and 0.15.

**Fig 1:**
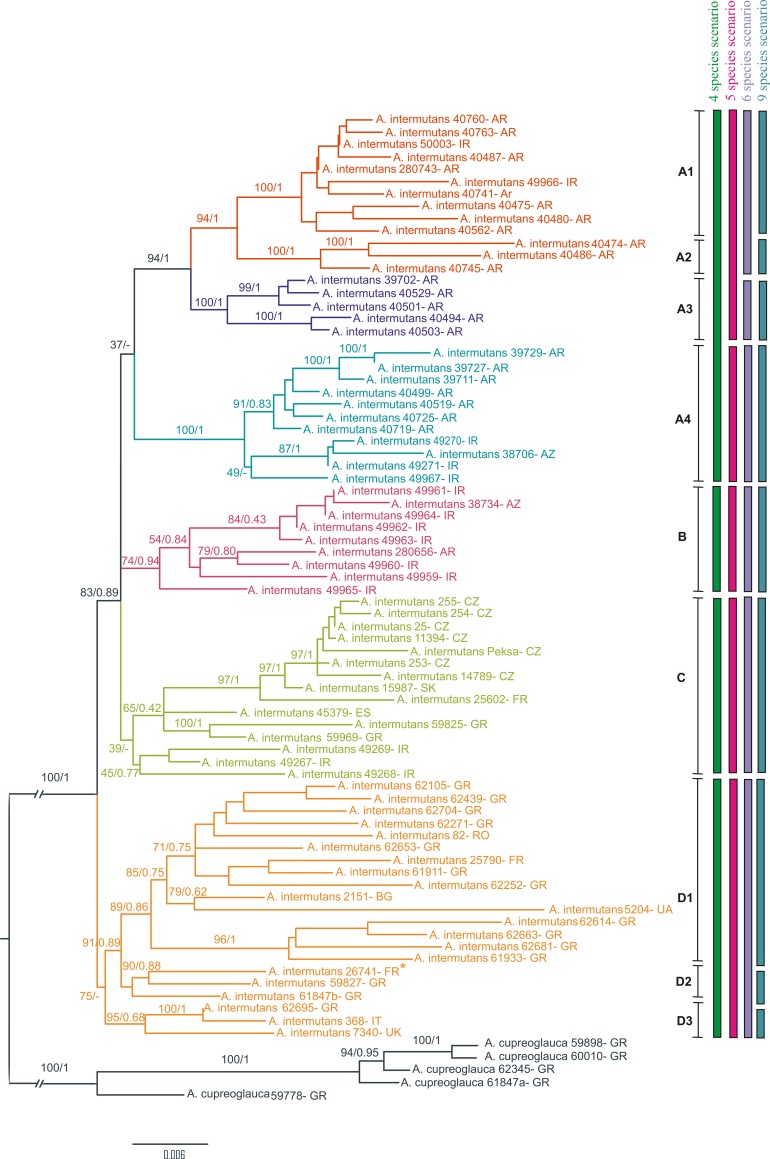
Concatenated gene tree. Maximum likelihood tree (IQ tree analysis) showing phylogenetic relationships among *Aspiciliella intermutans* candidate species based on the ITS, nrLSU, MCM7 and mtSSU concatenated dataset. Bootstrap values and Bayesian posterior probabilities (BS/PP) are shown above their respective branches. The six candidate species within the *A*. *intermutans* complex obtained in the species delimitation analysis based on BP&P and Bayes factor are highlighted by the different color. * = Sample collected at 160 km from the type locality.

Since the results of different single-locus species delimitation analyses were not congruent, we used the main monophyletic groups obtained from concatenated dataset analysis in IQ tree, as species scenarios for subsequent analyses. We tested a total of four different species delimitation scenarios: with four (A1+A2+A3+A4, B, C and D), five (A1+A2+A3, A4, B, C and D), six (A1+A2, A3, A4, B, C and D) and nine species (A1, A2, A3, A4, B, C, D1, D2 and D3) (Fig 1).

We used the coalescent-based hierarchical Bayesian model *BEAST implemented in BEAST v.1.8.0 to estimate species tree following our proposed species delimitation scenarios. *BEAST incorporates the coalescent process and the uncertainty associated with gene trees and nucleotide substitution model parameters and estimates the species tree directly from the sequence data [47].

Species assignments are required *a priori* for *BEAST analyses. To designate species in *BEAST, we used four different scenarios (4, 5, 6 and 9 species) for assigning individuals to a ‘candidate species’, based on well supported groups in the phylogenetic tree obtained from the concatenated dataset. We used lognormal distributions for the relaxed uncorrelated rates for all loci, we selected a Yule process and gamma-distributed population sizes for the species-tree prior and a piecewise linear population size model with a constant root. Default values were used for remaining priors. Two independent Markov chain Monte Carlo (MCMC) analyses were run for a total of 100 million generations, sampling every 1000^th^ generations and excluding the 12500 trees as burn-in. We assessed the MCMC convergence and determined burn-ins by examining ESS values and likelihood plots in the program Tracer version 1.6. The posterior probabilities of nodes were computed from the sampled trees (excluding burn-in samples) using TreeAnnotator 1.8.0 [47].

We used the multispecies coalescent model implemented in the program BP&P v.3.2 [46] to estimate the posterior probability for each species scenario (4, 5, 6 and 9 species). BP&P incorporates coalescent theory and phylogenetic uncertainty into parameter estimation; and the posterior distribution for species delimitation models is sampled using a reversible-jump Markov Chain Monte Carlo (rjMCMC) method. We used the unguided species delimitation algorithm [48]. This algorithm explores different species delimitations and different species phylogenies, with fixed specimen assignments to populations. The program attempts to merge populations into one species and uses the nearest neighbour interchange (NNI) or subtree pruning and regrafting (SPR) algorithms to change the species tree topology [46]. We performed analyses using algorithms 0 and 1 using three prior theta (θ: is a parameter that is proportional to the product of the population size and the mutation rate) assigned to gamma distributions of G (2, 10), G (2, 100) and G (2, 1000), combined with root age (τ0) assigned G (2, 2000). Rates were allowed to vary among loci (locus rate = 1), and the analyses were set for automatic fine-tune adjustments. Each reversible-jump Monte Carlo (rjMCMC) analysis was run for 100,000 generations, sampling each generation and specified a burn-in of the first 8 000 generations. Each analysis was run twice to confirm consistency between runs. Speciation probabilities greater than 0.95 were considered as supported species delimitations.

Since different species delimitation analyses supported different scenarios for the *Aspiciliella intermutans* complex, the most likely hypothesis of species boundaries was assessed using Bayes factor delimitation (BFD) test [49]. Bayes factor uses a Bayesian coalescent-based reconstruction of species trees to compare the different species delimitation models, calculating the marginal likelihood and does not require a guide tree. We calculated marginal likelihood estimates (MLEs) for four species delimitation scenarios (4, 5, 6 and 9) species. For each scenario, we reconstructed a species tree using *BEAST v.1.8.0. The analyses were run with the same substitution models used in MrBayes, a birth-death model for the species tree prior; population size model set to piecewise linear and constant root. The MCMC chain was run for 20,000,000 generations, sampled every 1000^th^, and the first 25% of each run was discarded as burn-in. MLEs were estimated using path sampling (PS) and stepping-stone sampling (SS) in *BEAST, with 100 path steps, a chain length of 100,000 generations and likelihoods saved every 100^th^ generations. The Bayes factor was calculated as 2 × (marginal likelihood of the best model (model1)—marginal likelihood of alternative model (model2). Positive values of Bayes factor support the best model against alternative models.

## Results

### Phylogenetic analyses

We generated a seven-locus data matrix consisting of 3205 aligned nucleotide positions. The matrix of the data sets had 183, 231, 139, 172, 1073, 507 and 900 unambiguously aligned nucleotide positions for Group I intron, ITS1, 5.8s, ITS2, nuLSU, MCM7 and mtSSU respectively. Table 2 summarized the best-fitting models of evolution for each locus. The multilocus data set was based on 79 individuals. A total of 218 new DNA sequences (ITS, nuLSU, mtSSU and MCM7) were generated for this study and were aligned with sequences obtained from GenBank (Table 3). The partitioned ML analysis of the concatenated data matrix yielded the optimal tree with Ln likelihood value = -12042.961 The mean LnL value of the two parallel runs of the Bayesian analysis for the seven combined loci was -12512.24 with a standard deviation of ±2.17.

**Table 3:** Voucher specimens and NCBI GenBank accession numbers of the ITS1-5.8S-ITS2, nrLSU, mtSSU and MCM7 sequences used in the phylogenetic analyses. New sequences are in bold.

Taxon	Country, Province, Voucher	nrLSU	mtSSU	ITS	MCM7
*A*. *cupreoglauca*	Greece, North Aegean, *Sipman & Raus 61847* (B)	KY576954	KY576930	KY618843	**—-**
*A*. *cupreoglauca*	Greece, North Aegean, *Sipman & Raus 62345* (B)	KY576955	KY576931	KY618844	**—-**
*A*. *cupreoglauca*	Greece, Cyclades Archipelago, *Sipman & Raus* 59778 (B)	**MH290743**	**MH349000**	**MH290788**	**—-**
*A*. *cupreoglauca*	Greece, Cyclades Archipelago, *Sipman 59898* (B)	**MH248842**	**MH248890**	**—-**	**—-**
*A*. *cupreoglauca*	Greece, Cyclades Archipelago, *Sipman 60010* (B)	**MH248843**	**MH248891**	**MH255584**	**—-**
*A*. *intermutans*	Iran, West Azerbaijan, *Zakeri 49267* (GLM)	KY576020	KY576019	KY596018	**—-**
*A*. *intermutans*	Iran, Ardebil, *Zakeri 49268* (GLM)	KY576943	KY576919	KY596005	**—-**
*A*. *intermutans*	Iran, Ardebil, *Zakeri 49269* (GLM)	KY576949	KY576925	KY596013	**—-**
*A*. *intermutans*	Iran, West Azerbaijan, *Zakeri 49270* (GLM)	KY576950	KY576926	KY596014	**—-**
*A*. *intermutans*	Iran, West Azerbaijan, *Zakeri 49271* (GLM)	KY576951	KY576927	KY596015	**—-**
*A*. *intermutans*	Armenia, Vayots Dzor, *Zakeri 39727* (GLM)	KY576941	KY576917	KY596006	**—-**
*A*. *intermutans*	Armenia, Vayots Dzor, *Zakeri 39729* (GLM)	KY576942	KY576918	KY596007	**—-**
*A*. *intermutans*	Armenia, Aragatsotn, *Zakeri 40474* (GLM)	KY576944	KY576920	KY596008	**—-**
*A*. *intermutans*	Armenia, Aragatsotn, *Zakeri 40494* (GLM)	KY576945	KY576921	KY596009	**—-**
*A*. *intermutans*	Armenia, Ararat, *Zakeri 40501* (GLM)	KY576946	KY576922	KY596010	**—-**
*A*. *intermutans*	Armenia, Ararat, *Zakeri 40503* (GLM)	KY576947	KY576923	KY596011	**—-**
*A*. *intermutans*	Armenia, Ararat, *Zakeri 40719* (GLM)	KY576948	KY576924	KY596012	**—-**
*A*. *intermutans*	Azerbaijan, Lerik, *Otte 38706* (GLM)	KY576952	KY576928	KY596016	**—-**
*A*. *intermutans*	Greece, North Aegean, *Sipman & Raus 61911* (B)	KY576958	KY576935	KY618848	**—-**
*A*. *intermutans*	Greece, North Aegean, *Sipman & Raus 62681* (B)	KY576960	KY576937	KY618850	**—-**
*A*. *intermutans*	Greece, North Aegean, *Sipman & Raus 62695* (B)	**MH257199**	**MH257235**	**MH210647**	**MH257270**
*A*. *intermutans*	Greece, North Aegean, *Sipman & Raus 61847* (B)	**MH257198**	**MH257234**	**MH210648**	**MH257261**
*A*. *intermutans*	Greece, North Aegean, *Sipman & Raus 62653* (B)	**MH257200**	**MH257236**	**MH210649**	**MH257262**
*A*. *intermutans*	Greece, North Aegean, *Sipman & Raus 62704* (B)	**MH257201**	**MH257237**	**MH210650**	**MH257263**
*A*. *intermutans*	Greece, North Aegean, *Sipman & Raus 62252* (B)	**MH257202**	**MH257238**	**MH210651**	**MH257264**
*A*. *intermutans*	Greece, North Aegean, *Sipman & Raus 62271* (B)	**MH257203**	**MH257239**	**MH210652**	**MH257271**
*A*. *intermutans*	Greece, North Aegean, *Sipman & Raus 62105* (B)	**MH257204**	**MH257240**	**MH210653**	**MH257265**
*A*. *intermutans*	Greece, North Aegean, *Sipman & Raus 62439* (B)	**MH257205**	**MH257241**	**MH210654**	**MH257272**
*A*. *intermutans*	Greece, North Aegean, *Sipman & Raus 62614* (B)	**MH257206**	**MH257242**	**MH210655**	**MH257273**
*A*. *intermutans*	Greece, North Aegean, *Sipman & Raus 62663* (B)	**MH257207**	**MH257243**	**MH210656**	**MH257274**
*A*. *intermutans*	Greece, North Aegean, *Sipman & Raus 61933* (B)	**MH257208**	**MH257244**	**MH210657**	**MH257266**
*A*. *intermutans*	Armenia, Vayots Dzor, *Zakeri 39702* (GLM)	**—-**	**MH257209**	**MH210658**	**MH257267**
*A*. *intermutans*	Armenia, Vayots Dzor, *Zakeri 39711* (GLM)	**—-**	**MH257210**	**MH210659**	**MH257245**
*A*. *intermutans*	Armenia, Aragatsotn, *Zakeri 40475* (GLM)	**MH257184**	**MH257211**	**MH210660**	**MH257246**
*A*. *intermutans*	Armenia, Aragatsotn, *Zakeri 40480* (GLM)	**MH257185**	**MH257212**	**MH210661**	**MH257268**
*A*. *intermutans*	Armenia, Aragatsotn, *Zakeri 40486* (GLM)	**MH257186**	**MH257213**	**MH210662**	**—-**
*A*. *intermutans*	Armenia, Aragatsotn, *Zakeri 40487* (GLM)	**MH257187**	**MH257214**	**MH210663**	**MH257247**
*A*. *intermutans*	Armenia, Ararat, *Zakeri 40499* (GLM)	**MH257188**	**MH257215**	**MH210664**	**MH257248**
*A*. *intermutans*	Armenia, Ararat, *Zakeri 40519* (GLM)	**—-**	**MH257216**	**MH210665**	**—-**
*A*. *intermutans*	Armenia, Ararat, *Zakeri 40529* (GLM)	**MH257189**	**MH257217**	**MH210666**	**—-**
*A*. *intermutans*	Armenia, Aragatsotn, *Zakeri 40562* (GLM)	**MH257190**	**MH257218**	**MH210667**	**MH257249**
*A*. *intermutans*	Armenia, Ararat, *Zakeri 40725* (GLM)	**—-**	**MH257219**	**MH210668**	**—-**
*A*. *intermutans*	Armenia, Aragatsotn, *Zakeri 40741* (GLM)	**MH257191**	**MH257220**	**MH210669**	**MH257250**
*A*. *intermutans*	Armenia, Aragatsotn, *Zakeri 40745* (GLM)	**MH257192**	**MH257221**	**MH210670**	**—-**
*A*. *intermutans*	Iran, Ardebil, *Zakeri 40760* (GLM)	**—-**	**MH257222**	**MH210671**	**MH257269**
*A*. *intermutans*	Armenia, Aragatsotn, *Zakeri 40763* (GLM)	**MH257193**	**MH257223**	**MH210672**	**MH257251**
*A*. *intermutans*	Iran, Ardebil, *Zakeri 49965* (GLM)	**MH257194**	**MH257224**	**MH210673**	**MH257252**
*A*. *intermutans*	Iran, West Azerbaijan, *Zakeri 49967* (GLM)	**—-**	**MH257225**	**MH210674**	**MH257253**
*A*. *intermutans*	Iran, Ardebil, *Zakeri 49960* (GLM)	**MH257195**	**MH257226**	**MH210675**	**MH257254**
*A*. *intermutans*	Iran, Ardebil, *Zakeri 49959* (GLM)	**MH257196**	**MH257227**	**MH210676**	**—-**
*A*. *intermutans*	Iran, Ardebil, *Zakeri 49966* (GLM)	**—-**	**MH257228**	**MH210677**	**MH257256**
*A*. *intermutans*	Iran, Khorasan Razavi, *Zakeri 49964* (GLM)	**—-**	**MH257229**	**MH210678**	**MH257257**
*A*. *intermutans*	Iran, Khorasan Razavi, *Zakeri 49961* (GLM)	**—-**	**MH257230**	**MH210679**	**MH257258**
*A*. *intermutans*	Azerbaijan, Lerik, *Otte 38734* (GLM)	**MH257197**	**MH257231**	**MH210680**	**—-**
*A*. *intermutans*	Iran, Khorasan Razavi, *Zakeri 49963* (GLM)	**—-**	**MH257232**	**MH210681**	**MH257259**
*A*. *intermutans*	Iran, Khorasan Razavi, *Zakeri 49962* (GLM)	**—-**	**MH257233**	**——**	**MH257260**
*A*. *intermutans*	Iran, Golestan, *Zakeri 50003* (GLM)	**—-**	**—-**	**—-**	**MH257255**
*A*. *intermutans*	Romania, Măcin Mts, *J*. *Šoun* 82 (hb. Šoun)	**MH248846**	**MH248875**	**—-**	**—-**
*A*. *intermutans*	Slovakia, Cerová vrchovina, *Z*. *Palice 15987* (PRA)	**MH248857**	**MH248871**	**MH255575**	**MH293579**
*A*. *intermutans*	France, Provence-Alpes-Côte d'Azur, *C*. *Roux 25790* (CR)	**MH248863**	**MH248869**	**MH255576**	**MH293580**
*A*. *intermutans*	France, Languedoc-Roussilon, *C*. *Roux 25602* (CR)	**MH248849**	**MH248870**	**—-**	**MH293588**
*A*. *intermutans*	France, Bretagne, *C*. *Roux 26741* (CR)	**MH248847**	**MH248874**	**MH255573**	**MH293576**
*A*. *intermutans*	Armenia, Vayots Dzor province, *M*. *Oganesian* (GZU280656)	**MH248845**	**MH248877**	**MH255572**	**MH293590**
*A*. *intermutans*	Armenia, Shirak province, *S*. *Harutyunyan & H*. *Mayrhofer* (GZU280743)	**MH248858**	**MH248878**	**—-**	**MH293591**
*A*. *intermutans*	Spain, Prov. Zamora, *H*. *Sipman 45379* (B)	**MH248844**	**MH248868**	**—-**	**MH293582**
*A*. *intermutans*	Italy, Sardinia, *N*.*M*. *Nöske 368* (B)	**MH248862**	**MH248879**	**—-**	**MH293583**
*A*. *intermutans*	Greece, Cyclades Archipelago, *H*. *Sipman 59825* (B)	**MH248859**	**MH248882**	**MH255571**	**MH293570**
*A*. *intermutans*	Greece, Cyclades Archipelago, *H*. *Sipman 59827* (B)	**MH248860**	**MH248881**	**MH255569**	**MH293571**
*A*. *intermutans*	Greece, Cyclades Archipelago, *H*. *Sipman 59969* (B)	**MH248861**	**MH248873**	**MH255570**	**MH293572**
*A*. *intermutans*	Czech Republic, Otvovická skála, *O*. *Peksa* (PL)	**MH248850**	**MH248889**	**—-**	**MH293592**
*A*. *intermutans*	Ukraine, Pervomaysk, *J*. *Vondrák 7340* (PRA)	**MH248848**	**MH248876**	**MH255567**	**MH293573**
*A*. *intermutans*	Ukraine, Mikolajivska oblast, *J*. *Vondrák 5204* (PRA)	**—-**	**MH248880**	**MH255577**	**MH293574**
*A*. *intermutans*	Bulgaria, Eastern Rodopi Mts, *J*. *Vondrák 2151* (PRA)	**MH248865**	**MH248872**	**MH255568**	**MH293575**
*A*. *intermutans*	Czech Republic, Central Bohemia, *V*. *Lenzová 255* (PRC)	**MH248854**	**MH248885**	**MH255579**	**MH293593**
*A*. *intermutans*	Czech Republic, Central Bohemia, *V*. *Lenzová 254* (PRC)	**MH248851**	**MH248886**	**MH255578**	**MH293589**
*A*. *intermutans*	Czech Republic, Central Bohemia, *V*. *Lenzová 253* (PRC)	**MH248856**	**MH248883**	**MH255582**	**MH293587**
*A*. *intermutans*	Czech Republic, Central Bohemia, *V*. *Lenzová 25* (PRC)	**MH248852**	**MH248887**	**MH255581**	**MH293577**
*A*. *intermutans*	Czech Republic, Central Bohemia, *Z*. *Palice 14789* (PRA)	**MH248853**	**MH248884**	**MH255583**	**MH293569**
*A*. *intermutans*	Czech Republic, Central Bohemia, *Z*. *Palice 11394* (PRA)	**MH248855**	**MH248888**	**MH255580**	**MH293578**

Since the topologies of the trees estimated from Bayesian methods and ML did not have any well-supported conflict, only ML topologies from IQ tree are shown with bootstrap and posterior probability values indicated on the nodes (Fig 1). In the concatenated tree topology, specimens of *A*. *intermutans* were recovered in several distinct clades (named hereafter A, B, C and D), in some cases with low support especially in Bayesian analyses (Fig 1). In all the groups, subclades with a strong support were also found.

#### Species delimitation analyses

Our exploratory analyses showed that the single gene tree were not congruent. Further, ITS region was resulted too variable and the nuLSU too conserved in the *A*. *intermutans* group (results not shown). The monophyletic groups B, C, D1, D2 and D3 obtained in the concatenated tree (Fig 1) were not reciprocally monophyletic in the single gene trees. The clades A1, A2, A3 and A4 were recovered in mtSSU gene tree and A3 in MCM7 gene tree (see S1 Fig and S2 Fig).

ABGD analyses applied to MCM7 and mtSSU dataset detected 12 and 9 candidate species respectively. The results of these analyses are shown in supporting information.

The results of the multispecies coalescent species delimitation method BP&P with different algorithms 0 and 1 are summarized in Tables 4 and 5. The two algorithms in BP&P consistently distinguished 4–6 independent evolutionary lineages. While the 4, 5 and 6 species topologies had a speciation probability of >0.96 threshold in the runs with algorithm 1 and 0 (values ranged between 0.96–1.0), the nine species topology was only supported (0.95) with algorithm 1 and a prior distribution on ancestral population size (theta) with a gamma distribution of G (2, 1000).

**Table 4:** Species delimitations (excluding outgroup) and their posterior probabilities.

Species scenarios	Ancestral population sizes (theta)					
	Algorithm 0			Algorithm 1		
	G (2,10)	G (2–100)	G (2,1000)	G (2,10)	G (2,100)	G (2,1000)
**4-species**	**0.99**	**0.99**	**1.00**	**0.99**	**1.00**	**0.96**
**5-species**	**0.99**	**1.00**	**1.00**	**0.99**	**0.99**	**1.00**
**6-species**	**0.99**	**1.00**	**1.00**	**0.99**	**1.00**	**1.00**
**9-species**	0.38	0.67	0.82	0.59	0.71	**0.95**

Prior distributions on ancestral population sizes (theta) assigned to different gamma distributions (G) combined with root age (tau) assigned to G (2, 2000).

**Table 5:** Delimited species and their posterior probabilities.

Species scenarios		Algorithm 0			Algorithm 1		
		G (2,10)	G (2,100)	G (2,1000)	G (2,10)	G (2,100)	G (2,1000)
**4-species**	**A**	**1.00**	**1.00**	**1.00**	**1.00**	**1.00**	**1.00**
	**B**	**0.99**	**0.99**	**1.00**	**0.99**	**1.00**	**0.99**
	**C**	**0.99**	**0.99**	**1.00**	**0.99**	**1.00**	**0.99**
	**D**	**0.99**	**1.00**	**1.00**	**1.00**	**1.00**	**1.00**
	**outgroup**	**0.99**	**1.00**	**1.00**	**1.00**	**1.00**	**1.00**
	**D+outgroup**	**0.0001**	**-**	**-**	**-**	**-**	**-**
	**B+C**	**0.00006**	**0.00001**	**-**	**0.0019**	**-**	**0.03**
**5-species**	**A1+A2+A3**	**1.00**	**1.00**	**1.00**	**1.00**	**1.00**	**1.00**
	**A4**	**1.00**	**1.00**	**1.00**	**1.00**	**1.00**	**1.00**
	**B**	**0.99**	**1.00**	**1.00**	**0.99**	**0.99**	**1.00**
	**C**	**0.99**	**1.00**	**1.00**	**0.99**	**0.99**	**1.00**
	**D**	**1.00**	**1.00**	**1.00**	**1.00**	**1.00**	**1.00**
	**outgroup**	**1.00**	**1.00**	**1.00**	**1.00**	**1.00**	**1.00**
	**B+C**	**0.0011**	**-**	**-**	**0.0023**	**0.00007**	**-**
**6-species**	**A1+A2**	**1.00**	**1.00**	**1.00**	**1.00**	**1.00**	**1.00**
	**A3**	**0.99**	**1.00**	**1.00**	**0.99**	**1.00**	**1.00**
	**A4**	**0.99**	**1.00**	**1.00**	**0.99**	**1.00**	**1.00**
	**B**	**0.99**	**1.00**	**1.00**	**0.99**	**1.00**	**1.00**
	**C**	**0.99**	**1.00**	**1.00**	**0.99**	**1.00**	**1.00**
	**D**	**1.00**	**1.00**	**1.00**	**1.00**	**1.00**	**1.00**
	**outgroup**	**1.00**	**1.00**	**1.00**	**1.00**	**1.00**	**1.00**
	**B+C**	**0.0016**	**-**	**-**	**0.0018**	**-**	**-**
	**A4+C**	**0.0008**	**-**	**-**	**0.0009**	**-**	**-**
**9-species**	**A1**	**1.00**	**1.00**	**0.99**	**0.99**	**0.99**	**1.00**
	**A2**	**0.97**	**0.99**	**0.99**	**0.96**	**0.99**	**0.99**
	**A3**	**1.00**	**0.99**	**1.00**	**0.99**	**0.99**	**1.00**
	**A4**	**0.98**	**0.99**	**0.99**	**0.98**	**0.99**	**1.00**
	**B**	**0.92**	**0.99**	**1.00**	**0.98**	**0.99**	**1.00**
	**C**	**0.65**	**0.99**	**0.99**	**0.95**	**0.99**	**0.97**
	**D1**	**0.99**	**0.77**	**0.89**	**0.97**	**0.88**	**1.00**
	**D2**	**0.68**	**0.67**	**0.83**	**0.63**	**0.71**	**0.95**
	**D3**	**0.40**	**0.89**	**0.96**	**0.62**	**0.83**	**0.95**
	**outgroup**	**1.00**	**0.99**	**0.99**	**1.00**	**0.99**	**0.99**
	**D1+D2**	**-**	**0.22**	**0.13**	**0.01**	**0.11**	**-**
	**D2+D3**	**0.20**	**0.09**	**0.03**	**0.29**	**0.16**	**0.02**
	**C+D3**	**0.29**	**0.001**	**0.0005**	**0.01**	**0.005**	**0.0009**
	**A3+A2**	**-**	**0.0006**	**-**	**0.0007**	**0.00005**	**-**
	**A4+A2**	**0.01**	**0.0005**	**0.0003**	**0.01**	**0.0009**	**-**
	**B+D3**	**0.006**	**0.0003**	**-**	**0.01**	**0.0003**	**-**
	**D1+B**	**0.003**	**0.0003**	**-**	**0.004**	**-**	**-**
	**C+D2+B**	**0.05**	**0.0002**	**0.00003**	**0.03**	**0.001**	**0.01**
	**C+D2**	**-**	**0.0001**	**0.0009**	**-**	**0.002**	**0.001**
	**C+B**	**0.001**	**0.0001**	**-**	**-**	**0.005**	**-**
	**A3+D3**	**-**	**0.00008**	**-**	**-**	**-**	**-**
	**B+D2**	**0.03**	**0.00007**	**0.0009**	**0.002**	**0.002**	**-**
	**D2+ outgroup**	**-**	**0.00003**	**-**	**-**	**0.00003**	**0.0005**
	**A2+D2**	**-**	**-**	**-**	**-**	**-**	**0.0004**

Prior distributions on ancestral population sizes (theta) assigned to different gamma distributions (G) combined with root age (tau) assigned to G (2, 2000).

**Fig 2:**
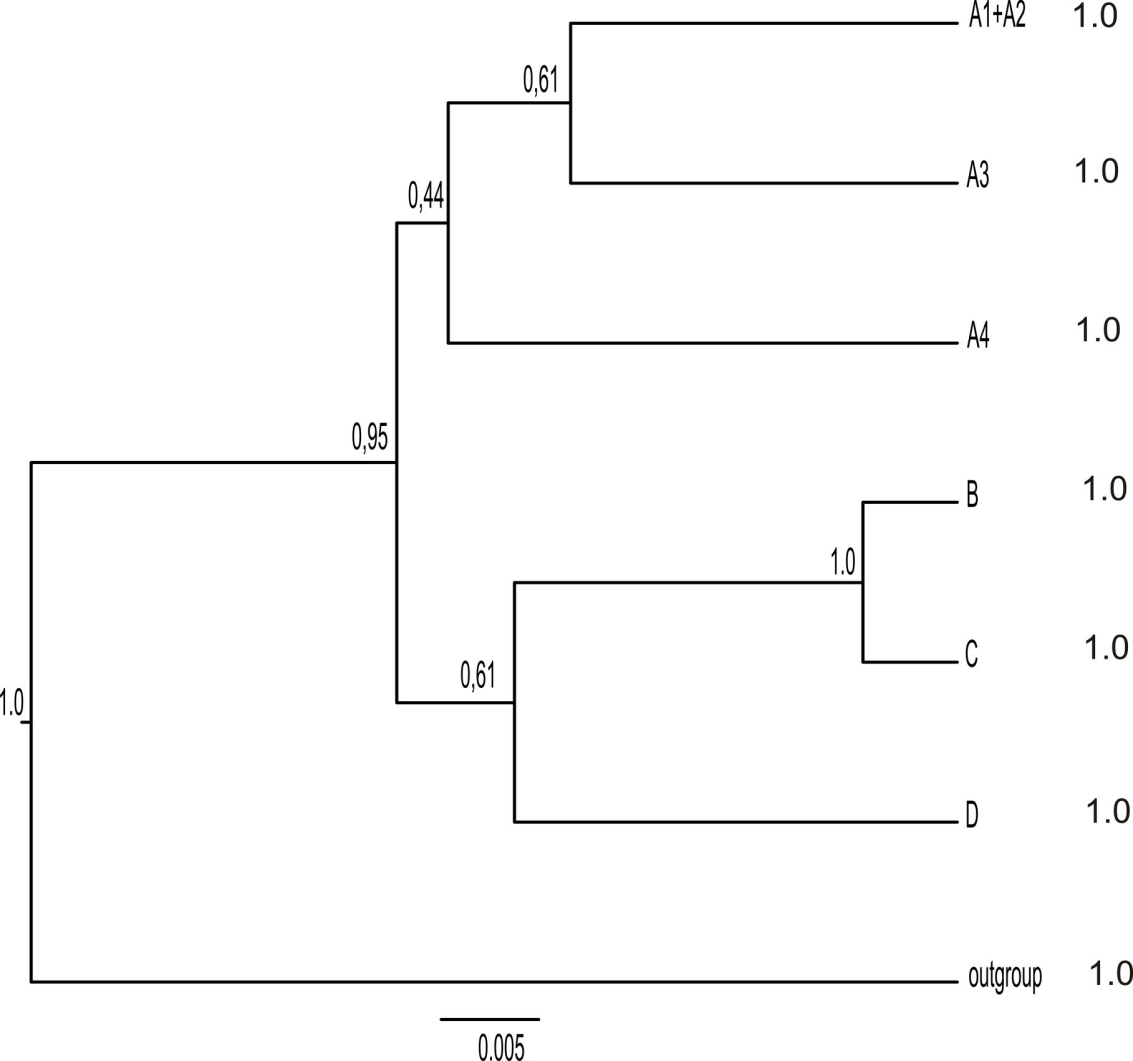
Species tree. Consensus *BEAST species tree of the *Aspiciliella intermutans* complex for six species scenario. Posterior probabilities at nodes indicate the support in the *BEAST analyses. The posterior probability of each delimited species calculated by BP&P is indicated in front of each putative species.

The Bayes factor delimitation results are provided in Table 6. BFD test supports the 6-species delimitation scenario model as the best scenario over other models tested, i.e. four, five and nine species delimitation scenarios. The 6-species scenario corresponds to the well supported clades: A1+A2, A3, A4, B, C and D. Clades C and D were not strongly supported in the concatenated gene tree. However, these were supported by the *BEAST species tree (Fig 2).

**Table 6:** Marginal likelihood and Bayes factor values for alternative species delimitation scenarios.

Model	Marginal Likelihood estimates (MLE)	MLE 6 species (model 1) -MLE i species (model 2)	2lnBF = 2 (MLE 6 species -MLE i species)
**9 species**	-13865,84647	129,0528333	258,1056665
**6 species**	**-13736,79364**	N/A	
**5 species**	-13765,60161	28,80797094	57,61594187
**4 species**	-13756,53862	19,74498099	39,48996197

Marginal-likelihood estimates and Bayes factor testing results (2lnBf) BF = 2 x (model1-model2); the model receiving the best marginal-likelihood score for each estimation method is indicated by a 2lnBf; score = N/A, and its associated marginal likelihood is in bold.

### Phenotypical and chemical examination of *Aspiciliella intermutans* samples

#### Chemistry

Two chemotypes were detected by TLC: 1) with norstictic acid and traces of connorstictic acid; 2) with norstictic, stictic and traces of cryptostictic acids. Similar results of a chemical analysis of *A*. *intermutans* from Greece were published by Sipman & Raus [50]. The second chemotype was only detected in some samples of the clade D, and the first chemotype was found in all clades. The two chemotypes detected inside the *A*. *intermutans* complex do not discriminate putative lineages.

#### Morphology

No distinct morphological features were observed to segregate candidate species, except for the areoles color and form. Clades A1+A2 and B are characterized by a thick thallus and larger areoles with a brown to red brown color, in contrast to the thinner thallus and smaller areoles with a gray to light brown color of the samples in clades A3 and A4. However, in samples from different parts of Europe, grouped in clades C and D, all these characters were totally intermixed. Although, the variability in the thallus form and color of the *A*. *intermutans* complex was considerable; these appeared more or less homogeneous in the samples from Iran and Armenia. Consequently, we tend to not consider them taxonomically relevant in this group.

### Substrate preferences and distribution

The species of the *Aspiciliella intermutans* complex occur on siliceous or volcanic rocks in open habitats. The species grow on substrates with mesic (e.g. basalt) to low pH (e.g. andesite, granite and quartzite rocks). They often grow on exposed, more or less horizontal faces of outcrops. The clades C and D appear to have broad distributions, collected from geographically different regions; clades A1, A2, A3, A4 and B consisted only of specimens collected in western north Iran, Armenia and Azerbaijan.

## Discussion

The frequent occurrence of cryptic species has been shown in macrolichen species complexes, examples include the *Cladia* aggregate [25], *Dictyonema glabratum* (Spreng.) D. Hawksw. [51], *Letharia columbiana* (Nutt.) J. W. Thomson [52], *Parmelia saxatilis* (L.) Ach. [53], *Parmotrema reticulatum* (Del-Prado et al. 2016), *Pectenia atlantica* (Degel.) P. M. Jørg., L. Lindblom, Wedin & S. Ekman [54] and *Rhizoplaca melanophthalea* [22]. However, this phenomenon has poorly been described in microlichen species complexes (see e.g. *Graphis scripta* (L.) Ach.) [55]. This could most probably be due to less attention paid to microlichens or the thought that microlichen species usually encompass differential features, especially ascomata.

Our study uncovers cryptic species diversity in the widespread Mediterranean microlichen species, *Aspiciliella intermutans*, and adds another example of species complexes to the microlichens. We show that *A*. *intermutans* as currently described is monophyletic but include several cryptic lineages. Our multispecies coalescent species delimitation approaches and BFD test supported the occurrence of at least six candidate species in the *A*. *intermutans* complex. Our results are in the accordance with a previous study showing different genetic clusters within the *A*. *intermutans* group [5].

The results of phylogenetic analyses of single locus datasets were incongruent in *A*. *intermutans* group, suggesting incomplete lineage sorting in the loci or ongoing gene flow or long generation times. These dramatically increase the amount of time required for a species to be recognized under a strict monophyly criterion [56]. The phylogenetic species delimitation criteria such as reciprocal monophyly has been widely used to recognize species however, this criterion may fail to accurately delimit species boundaries in recently diverged species [57]. Multispecies coalescent-based approaches offer a statistic framework for testing species boundary in group with recent diversification histories and does not require species to be reciprocally monophyletic in single gene trees [58,59].

Since the results of single locus datasets were inconclusive, a multilocus data set from *A*. *intermutans* samples was analyzed in multispecies coalescent-based approaches. Mitochondrial SSU tree topology recovered most lineages identified in the combined analyses, suggesting that a strong phylogenetic signal from mtSSU may have been dominating the analysis of the concatenated genes. The distinctiveness of the different monophyletic clades in the concatenated phylogenetic tree is clear but phylogenetic analyses alone are insufficient (lack of a strong support in some clades) to draw conclusions on species boundaries in this complex. Therefore, we used a combination of methods to address the species delimitation problems in this complex.

Although the species delimitation analyses indicated the presence of different species lineages in the *A*. *intermutans* complex, we followed a conservative approach as suggested by *BEAST and BP&P, which is sensitive enough to detect evolutionarily distinct lineages at very shallow timescales [60]. Based on all of the available evidence, including the multispecies coalescent-based species delimitation inferences *BEAST, BP&P, and Bayes Factor test, we could objectively circumscribe six candidate species within the *A*. *intermutans* complex.

We also examined chemistry, morphology, substrate preferences and geographic distribution of the samples, in order to correlate with tree topology. Generally, we found little corroboration between morphological and ecological characters with our candidate species, and also secondary metabolite data provided only a very limited support for the putative species. However, that is a case as in other cryptic lineages of lichenized-fungi. The presence of cryptic species in lichen-forming fungi without any recognizable phenotypical characterization of the clades has been demonstrated in some studies [61].

We did not try to extract DNA of the *A*. *intermutans* type material from St. Laon [10], because the material was too old to obtain a sequence. However, we obtained DNA from an *A*. *intermutans* sample (France, Bretagne, *C*. *Roux 26741*) collected 160 km northwest of St. Laon, and the sample is grouped in the clade D together with samples from other parts of Europe. We suggest the candidate species D (clade D) as the putative species for *A*. *intermutans* s. str. and all other clades as putative different cryptic species for *A*. *intermutans* complex.

We know that all published taxa with the characteristics of *Aspiciliella* should eventually be compared with the species distinguishable in this group in order to recognize any names with priority or synonyms. However, this should be the last step after the taxa have been convincingly identified and circumscribed in a manner that those older type specimens can be doubtlessly affiliated to them. Currently, our study suggests that it is not possible to delimit such taxa convincingly, and therefore it is not possible for us on the moment to decide about valid taxonomic names.

### Characters of the putative species in the *Aspiciliella intermutans* complex

The phenotypic and geographic features corresponding to the different clades are summarized in Table 7.

**Table 7:** The phenotypic and geographic features corresponding to the different clades in the *Aspiciliella intermutans* complex recognized by BP&P and Bayes factor analyses on the concatenated phylogenetic tree.

Clades	Geographical region	No. of samples (74)	Altitude (m)	Substrate	Thallus form	Thallus color	Chemotypes
**A1+A2**	Armenia, Northwestern and North of Iran	13	1800–3044	Mostly basaltic and andesite rocks	Large and irregular areoles (0.3–2 mm)	Gray-brown to light-brown with pale marginal lines	1
**A3**	Armenia	5	1390–1850	Granite	Small and regular (0.3–1 mm)	Gray and pruinose	1
**A4**	Armenia, Northwestern Iran and Azerbaijan	11	1300–1600 (one sample 1900 m)	Andesite, quartz-andesite, granites	Small and regular (0.3–1 mm)	Gray and pruinose	1
**B**	Armenia, Northwestern and Northeastern Iran and Azerbaijan	9	1885–1992	Mostly quartz-lamprophyre and granites	Large and irregular areoles (0.3–2 mm)	Gray-brown to light-brown with pale marginal lines	1
**C**	Czech Republic, Slovakia, Greece, France, Spain and Northwestern Iran	15	0–1898	Mostly basalts and andesite	0.3–1.5 mm	Gray to dark-gray	1
**D**	Romania, Ukraine, Bulgaria, Italy, France and Greece (mostly)	21	2–760	Mostly granite	No distinctive features	No distinctive features	1 and 2

**Clades A1+A2** were recovered as a well-supported lineage (BS = 94/PP = 0.1), sister to the clade A3 with strong nodal support (BS = 94/PP = 0.1) they show some differences in ecological and morphological patterns. The samples of clade A1 developed a gray-brown to light-brown thallus with pale marginal lines and large (0.3–2 mm) and irregular areoles; some areoles come over others and show a superimposed structure. They are commonly found at medium to higher elevation (1800–3044 m) in the North and North-West of Iran and in Armenia.

**Clade A3** was recovered as a well-supported lineage (BS = 100/PP = 0.1). The samples develop a gray thallus, which is light brown with pale marginal lines at the edge of the thallus and in the middle totally gray and pruinose; areoles are smaller (0.3–1 mm) than samples in A1+A2 clades and they do not show an irregular structure. They are commonly found at lower to medium elevation (1390–1850 m) in Armenia.

**Clade A4** was recovered with a high nodal support (BS = 100/PP = 0.1) and includes samples morphologically similar to clade A3, but prefers substrates with lower pH. The samples were collected from Azerbaijan, Armenia and Northwest of Iran, at medium elevation (1300–1900 m).

**Clade B** was recovered with a low nodal support (BS = 74/PP = 0.89) and includes samples from Azerbaijan, Armenia, the Northwest and Northeast of Iran, at medium to high elevation (1885–1992 m). The samples are morphologically similar to those in clade A1, but prefer substrates with higher pH.

**Clade C** was recovered with a low nodal support (BS = 39) and it occurs mostly on basaltic rocks at low to medium elevations, 0–1030 m. It has been collected from the Czech Republic, Slovakia, Greece, France and Spain. It has been recorded also on andesite rocks, in medium to high elevations (1305–1898 m) in northwestern of Iran.

**Clade D** was recovered with a medium support (PP = 75) and it is sister to clade A-C with the remaining *Aspiciliella intermutans* samples (BS = 83/PP = 0.89). The samples have a wide morphological variation and they are from Romania, Ukraine, Bulgaria, Italy, France and mostly from Greece (North Aegean Region), at low elevations (2–760 m), mostly on granite rocks. Some samples in this clade show a chemotype with norstictic acid, stictic acid and a trace of cryptostictic acid, observed exclusively inside this clade

## Conclusions

**Fig 3:**
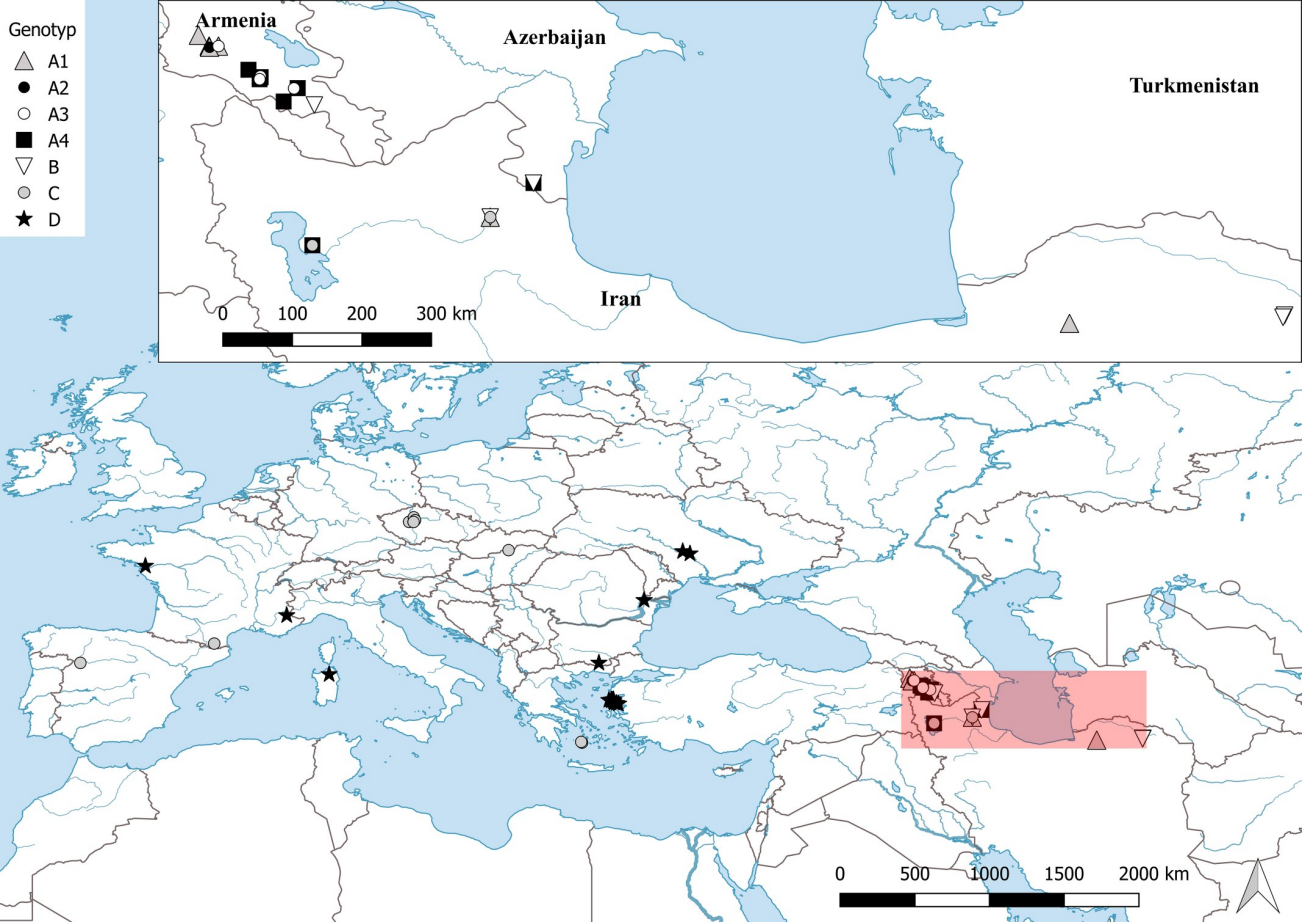
Geographical distribution. Geographical distribution of the *Aspiciliella intermutans* complex examined samples in this study. Symbols indicate the putative species.

Our study of the *Aspiciliella intermutans* species complex indicates that the genus *Aspiciliella* and the *A*. *intermutans* complex are more diverse in Eurasia than previously expected. Further, this study highlights the occurrence of cryptic species in microlichens. Our sampling was more intensive in Armenia, Iran, Greece and the eastern part of Europe however the samples from other distribution ranges of this species complex were either scarce or absent. While four of the six species-level lineages within the *A*. *intermutans* complex were restricted to Armenia and Iran, only two broadly distributed lineages were found in samples from Greece and also other parts of Europe (Fig 3). Based on our results we suggest that the Caucasian region could be the main biogeographical region for speciation in this complex.

Ultimately, rigorous sampling in other regions, such as Western and Central Europe, the Caucasian region, NE Iran, Turkmenistan Anatoly, Central Asia mountains (and also North America), will be needed to more accurately assess the distribution patterns of lineages within this group. Understanding the general geographic distribution of the candidate species identified in this study requires robust data from a broader geographic sampling, which may or may not correlate to additional lineages within the *A*. *intermutans* species-complex.

The authors plan a detailed revision of the *A*. *intermutans* species-complex in the near future, with more taxonomic, morphological and geographical sampling to characterize boundaries between the candidate species.

## Supporting information

S1 Fig mtSSU ML tree. ML phylogenetic tree (IQ tree analysis) of *Aspiciliella intermutans* complex from mtSSU sequences. Bootstrap values are shown above their respective branches. Our six candidate species within the *A*. *intermutans* complex based on BP&P and Bayes factor on the concatenated phylogenetic tree are highlighted by different colors as in Fig 1. The result of ABGD analyses for the mtSSU-single locus are shown in the picture.
(TIF)

S2 Fig MCM7 ML tree. ML phylogenetic tree (IQ tree analysis) of *Aspiciliella intermutans* complex from MCM7 sequences. Bootstrap values are shown above their respective branches. Our six candidate species within the *A*. *intermutans* complex based on BP&P and Bayes factor on the concatenated phylogenetic tree are highlighted by different colors as in Fig 1. The result of ABGD analyses for the MCM7-single locus are shown in the picture.
(TIF)

S1 Table Geographical distribution data. (ODS)

S1 File MCM7 sequence alignment. (TXT)

S2 File mtSSU sequence alignment. (TXT)

S3 File Sequence alignment of Concatenated gene tree. (TXT)

## References

[1] Nordin A, Savić S, Tibell L (2010) Phylogeny and taxonomy of *Aspicilia* and *Megasporaceae*. Mycologia 102: 1339–1349. 10.3852/09-266 20943564

[2] Nordin A (2015) New synonyms and lectotypes in Aspicilia (Megasporaceae, Ascomycota). Phytotaxa 192: 197–200.

[3] Nash THIII, Gries C, Bungartz F (eds.) 2007 (2008) Lichen Flora of the Greater Sonoran Region, Volume 3. Tempe, Arizona: Lichens Unlimited, Arizona State University. 575 pp. (with a 56-page insert containing 224 color photographs).

[4] Owe-Larsson B, Nordin A, Tibell L (2007) Aspicilia In: Nash TH III, Gries C, Bungartz F (eds) Lichen Flora of the greater Sonoran Desert region, lichens unlimited, volume 3 Arizona State University, Tempe Pp. 61–108.

[5] Zakeri Z, Divakar PK, Otte V (2017) Taxonomy and Phylogeny of *Aspiciliella*, a Resurrected genus of *Megasporaceae*, Including the New Species *A*. *portosantana*. Herzogia 30: 166–176.

[6] Zakeri Z, Gasparyan A, Aptroot A (2016) A new corticolous Megaspora (Megasporaceae) species from Armenia. Willdenowia 46: 245–251.

[7] Jaklitsch W, Baral HO, Lücking R, Lumbsch HT (eds) (2016) Syllabus of Plant Families Part 1/2 Ascomycota. Stuttgart: Borntraeger Science Publishers.

[8] Zakeri Z, Sipman H, Paukov A, Otte V (2019) Neotypification of Aspiciliella cupreoglauca and lectotypification and synonymization of Aspicilia reticulata (Megasporaceae, Ascomycota). Lichenologist 51 (1): 97–99.

[9] Werner RG (1932) Contribution á la flore cryptogamique du Maroc V. Cavanillesia 5: 157–174.

[10] Nylander W (1872) Addenda nova ad Lichenographiam Europaeam. Flora (Regensburg) 55: 353–365.

[11] Arnold FChG (1887) Lichenologische Ausflüge in Tirol. Verhandlungen der Zoologisch-Botanischen Gesellschaft in Wien 37: 81–150.

[12] Nimis P. L. & Martellos S. (2008) The Information System on Italian Lichens. Version 4.0. University of Trieste, Dept. of Biology, IN4.0/1 (http://dbiodbs.univ.trieste.it/).

[13] Llimona X, Hladun N (2001) Checklist of the lichens and lichenicolous fungi of the Iberian Peninsula and Balearic Islands. Bocconea 14: 5–581.

[14] Fletcher A, Purvis OW, Coppins BJ (2009) Aspicilia A. Massal Pp.: 181–188. In: Smith C. W., Aptroot A., Coppins B. J., Flechter A., Gilbert O. L., James P. W. & Wolseley P. A. (eds). The Lichens of Great Britain and Ireland. London: The British Lichen Society.

[15] Hafellner J (1995) A new checklist of lichens and lichenicolous fungi of insular Laurimacaronesia including a lichenological bibliography for the area. Fritschiana 5: 1–132.

[16] Pitard CJ, Bouly de Lesdain M (1909) Contribution à l'étude des lichens de Tunisie. Bulletin de la Sociète Botanique de France 56: 243–264.

[17] John V, Seaward MRD, Sipman HJM, Zedda L (2004) Lichens and lichenicolous fungi from Syria, including a first checklist. Herzogia 17: 157–177.

[18] Paukov AG, Frolov IV, Vondrakova OS (2014) New records of lichens of the genus *Aspicilia* in the Ural Mts. The Immanuel Kant Baltic Federal University Vestnik, Kaliningrad 7: 102–109.

[19] McCune B, Rosentreter R, Spribille T, Breuss O, Wheeler T (2014) Montana Lichens: An Annotated List. Monographs in North American Lichenology 2: 1–183.

[20] Del-Prado R, Divakar PK, Lumbsch HT, Crespo A (2016) Hidden genetic diversity in an asexually reproducing lichen forming fungal group. PLoS ONE 11: e0161031 10.1371/journal.pone.0161031 27513649 PMC4981466

[21] Leavitt SD, Esslinger TL, Nelsen MP, Lumbsch HT (2013a) Further species diversity in Neotropical Oropogon (Lecanoromycetes: Parmeliaceae) in Central America. Lichenologist 45: 553–564.

[22] Leavitt SD, Esslinger TL, Spribille T, Divakar PK, Lumbsch HT (2013b) Multilocus phylogeny of the lichen forming fungal genus Melanohalea (Parmeliaceae, Ascomycota): Insights on diversity, distributions, and a comparison of species tree and concatenated topologies. Molecular Phylogenetics and Evolution 66: 138–152. 23017822 10.1016/j.ympev.2012.09.013

[23] Leavitt SD, Fankhauser JD, Leavitt DH, Porter LD, Johnson LA, St Clair LL (2011a) Complex patterns of speciation in cosmopolitan ‘‘rock posy” lichens–Discovering and delimiting cryptic fungal species in the lichen–forming Rhizoplaca melanophthalma species–complex (Lecanoraceae, Ascomycota). Molecular Phylogenetics and Evolution 59: 587–602. 21443956 10.1016/j.ympev.2011.03.020

[24] Leavitt SD, Johnson LA, Goward T, St Clair LL (2011b) Species delimitation in taxonomically difficult lichen–forming fungi: An example from morphologically and chemically diverse Xanthoparmelia (Parmeliaceae) in North America. Molecular Phylogenetics and Evolution 60: 317–332. 10.1016/j.ympev.2011.05.012 21627994

[25] Parnmen S, Rangsiruji A, Mongkolsuk P, Boonpragob K, Nutakki A, Lumbsch HT (2012) Using phylogenetic and coalescent methods to understand the species diversity in the Cladia aggregata complex (Ascomycota, Lecanorales).–PLoS ONE 7: e52245 10.1371/journal.pone.0052245 23272229 PMC3525555

[26] Leavitt SD, Moreau CS, Lumbsch HT (2015) The Dynamic Discipline of Species Delimitation: Progress Toward Effectively Recognizing Species Boundaries in Natural Populations In: Upreti DK, Divakar PK, Shukla V, Bajpai R, editors. Recent Advances in Lichenology: Springer India p. 11–44.

[27] Lumbsch H T, Huhndorf SM (eds) (2007) Outline of Ascomycota. Myconet 13: 1–58.

[28] Orange A, James PW, White FJ (2001) Microchemical Methods for the Identification of Lichens London: British Lichen Society. ISBN 0-9540418-01.

[29] Park SY, Jang SH, Oh SO, Kim JA, Hur JS (2014) An easy, rapid, and cost-effective method for DNA extraction from various lichen taxa and specimens suitable for analysis of fungal and algal strains. Mycobiology 42: 311–316. 10.5941/MYCO.2014.42.4.311 25606001 PMC4298833

[30] Gardes M, Bruns TD (1993) ITS primers with enhanced specificity for *Basidiomycetes*–application to the identification of mycorrhizae and rusts. Molecular Ecology 2: 113–118. 8180733 10.1111/j.1365-294x.1993.tb00005.x

[31] White TJ, Bruns TD, Lee S, Taylor J (1990) Amplification and direct sequencing of fungal ribosomal RNA genes for phylogenetics Pp. 315–322. In: Innis M.A., Gelfand D.H., Sninsky J.J. & White T.J. (eds). PCR Protocols: A Guide to methods and Applications. New York: Academic Press.

[32] Vilgalys R, Hester M (1990) Rapid genetic identification and mapping of enzymatically amplified ribosomal DNA from several *Cryptococcus* species. Journal of Bacteriology 172: 4238–4246. 10.1128/jb.172.8.4238-4246.1990 2376561 PMC213247

[33] Zoller S, Scheidegger C, Sperisen C (1999) PCR primers for the amplification of mitochondrial small subunit ribosomal DNA of lichen-forming ascomycetes. Lichenologist 31: 511–516.

[34] Schmitt I, Crespo A, Divakar P, Fankhauser J, Herman-Sackett E (2009) New primers for promising single-copy genes in fungal phylogenetics and systematics. Persoonia 23: 35–40. 10.3767/003158509X470602 20198159 PMC2802727

[35] Leavitt SD (2010) Assessing traditional morphology- and chemistry-based species circumscriptions in lichenized ascomycetes: Character evolution and species delimitation in common western North American lichens. USA, Utah, Provo, Brigham Young University: Ph.D. dissertation.

[36] Katoh K., Asimenos G. & Toh H. 2009 Multiple Alignment of DNA Sequences with MAFFT. Methods in Molecular Biology 537: 39–64. 10.1007/978-1-59745-251-9_3 19378139

[37] Castresana J (2000) Selection of conserved blocks from multiple alignments for their use in phylogenetic analysis. Molecular Biology and Evolution 17: 540–553. 10.1093/oxfordjournals.molbev.a026334 10742046

[38] Nguyen LT, Schmidt HA, von Haeseler A, Minh BQ (2014) IQ‐TREE: a fast and effective stochastic algorithm for estimating maximum‐likelihood phylogenies. Molecular Biology and Evolution 32: 268–274. 10.1093/molbev/msu300 25371430 PMC4271533

[39] Chernomor O, von Haeseler A, Minh BQ (2016) Terrace aware data structure for phylogenomic inference from supermatrices. Systematic Biology. 65: 997–1008. 10.1093/sysbio/syw037 27121966 PMC5066062

[40] Kalyaanamoorthy S, Minh BQ, Wong TKF, von Haeseler A, Jermiin LS (2017) ModelFinder: fast model selection for accurate phylogenetic estimates. Nature Methods 14: 587–589. 10.1038/nmeth.4285 28481363 PMC5453245

[41] Minh BQ, Nguyen MA, von Haeseler A (2013) Ultrafast approximation for phylogenetic bootstrap. Molecular Biology and Evolution 30: 1188–1195 10.1093/molbev/mst024 23418397 PMC3670741

[42] Gutierrez Gabriel & Blanco Oscar & Divakar Pradeep& Lumbsch Thorsten & Crespo Ana. (2007). Patterns of Group I Intron Presence in Nuclear SSU rDNA of the Lichen Family Parmeliaceae. Journal of molecular evolution. 64 181–95. 10.1007/s00239-005-0313-y 17200806

[43] Ronquist F, Huelsenbeck JP (2003) MrBayes 3: Bayesian phylogenetic inference under mixed models. Bioinformatics 19: 1572–1574. 10.1093/bioinformatics/btg180 12912839

[44] Puillandre N, Lambert A, Brouillet S, Achaz G (2012) ABGD, Automatic Barcode Gap Discovery for primary species delimitation. Molecular Ecology 21: 1864–77. 10.1111/j.1365-294X.2011.05239.x WOS:000302616200008. 21883587

[45] Heled J, Drummond AJ (2010) Bayesian Inference of Species Trees from Multilocus Data. Molecular Biology and Evolution. 27: 570–80. 10.1093/molbev/msp274 19906793 PMC2822290

[46] Yang Z, Rannala B (2014) Unguided species delimitation using DNA sequence data from multiple loci. Molecular Biology and Evolution 31: 3125–3135. 10.1093/molbev/msu279 25274273 PMC4245825

[47] Drummond A J, Rambaut A (2007) BEAST. Bayesian evolutionary analysis by sampling trees. BMC Evolutionary Biology 7: 214 10.1186/1471-2148-7-214 17996036 PMC2247476

[48] Yang ZH (2015) The BPP program for species tree estimation and species delimitation. Current Zoology 61: 854–865.

[49] Grummer JA, Bryson RW, Reeder TW (2014) Species delimitation using Bayes factors: simulations and application to the Sceloporus scalaris species group (Squamata: Phrynosomatidae). Systematic Biology 63: 119–133. 10.1093/sysbio/syt069 24262383

[50] Sipman HJM, Raus T (2002) An inventory of the lichen flora of Kalimnos and parts of Kos (Dodecanisos, Greece). Willdenowia, 32(2):351–392

[51] Lücking R, Barrie FR, Genney D (2014) Dictyonema coppinsii, a new name for the European species known as Dictyonema interruptum (Basidiomycota: Agaricales: Hygrophoraceae), with a validation of its photobiont Rhizonema (Cyanoprokaryota: Nostocales: Rhizonemataceae). Lichenologist 46: 261–267.

[52] Altermann S, Leavitt SD, Goward T, Nelsen MP, Lumbsch HT (2014) How do you solve a problem like *Letharia*? A new look at cryptic species in lichen-forming fungi using Bayesian clustering and SNPs from multilocus sequence data. PLoS ONE 9: e97556 10.1371/journal.pone.0097556 24831224 PMC4022584

[53] Molina MC, Del-Prado R, Divakar PK, Sánchez-Mata D, Crespo A (2011) Another example of cryptic diversity in lichen-forming fungi: The new species Parmelia mayi (Ascomycota: Parmeliaceae). Organisms Diversity and Evolution 11: 331–342.

[54] Otálora MAG, Martínez I, Aragón G, Wedin M (2017) Species delimitation and phylogeography of the *Pectenia* species-complex: A misunderstood case of species-pairs in lichenized fungi, where reproduction mode does not delimit lineages. Fungal Biology 121: 222–233. 10.1016/j.funbio.2016.12.001 28215350

[55] Kraichak E, Lücking R, Aptroot A, Beck A, Dornes P, John, et al (2015) Hidden diversity in the morphologically variable script lichen (Graphis scripta) complex (Ascomycota, Ostropales, Graphidaceae). Organisms Diversity & Evolution 15: 447–458.

[56] Hudson RR, Coyne JA (2002) Mathematical consequences of the genealogical species concept. Evolution. 56: 1557 12353748 10.1111/j.0014-3820.2002.tb01467.x

[57] Knowles LL, Carstens BC (2007) Delimiting species without monophyletic gene trees. Systematic Biology. 56: 887–895. 10.1080/10635150701701091 18027282

[58] Camargo A, Morando M, Avila LJ, Sites JW (2012) Species delimitation with Abc and other coalescent-based methods: a test of accuracy with simulations and an empirical example with lizards of the Liolaemus Darwinii Complex (squamata: Liolaemidae). Evolution 66: 2834–2849. 10.1111/j.1558-5646.2012.01640.x 22946806

[59] Rannala B, Yang Z (2017) Efficient Bayesian species tree inference under the multispecies coalescent. Syst. Biol. 66: 823–842. 10.1093/sysbio/syw119 28053140 PMC8562347

[60] Yang Z, Rannala B (2010) Bayesian species delimitation using multilocus sequence data. Proceedings of the National Academy of Sciences of the United States of America 107:9264–9269. 10.1073/pnas.0913022107 20439743 PMC2889046

[61] Lumbsch HT, Leavitt SD (2011) Goodbye morphology? A paradigm shifts in the delimitation of species in lichenized fungi. Fungal Diversity 50: 59–72.

