# Supplementary figures and images for: Discovering cryptic species in the Aspiciliella intermutans complex (Megasporaceae, Ascomycota) – First results using gene concatenation and coalescent-based species tree approaches
Source: PLoS One. 2019 May 28;14(5):e0216675. doi: 10.1371/journal.pone.0216675 (PMC6538240; doi:10.1371/journal.pone.0216675)

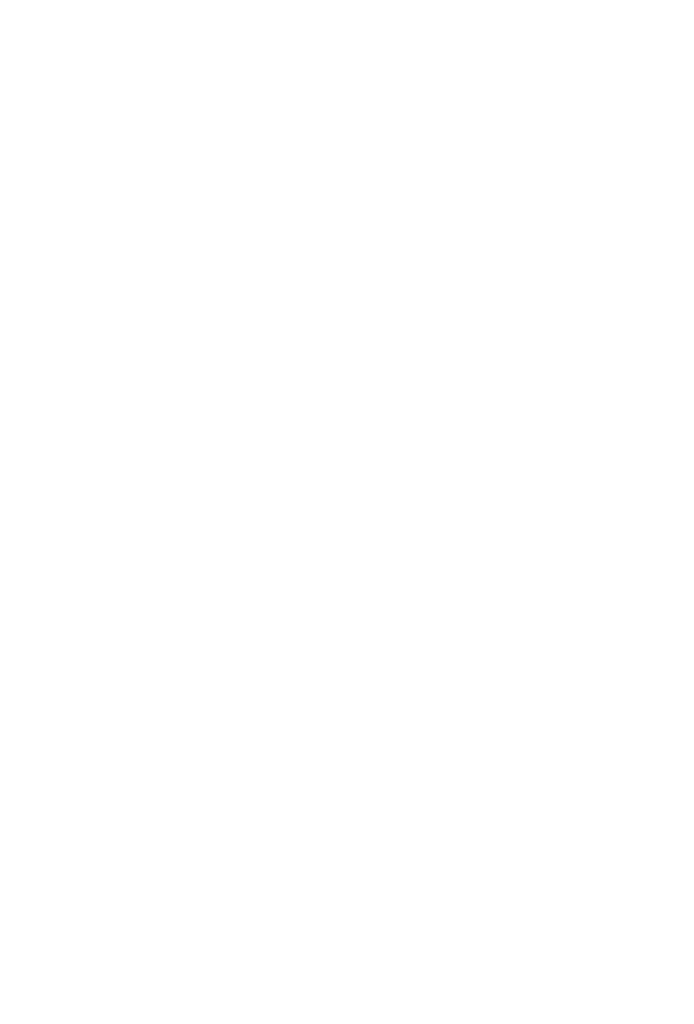

Supplement: S1 Fig — ML phylogenetic tree (IQ tree analysis) of Aspiciliella intermutans complex from mtSSU sequences. Bootstrap values are shown above their respective branches. Our six candidate species within the A. intermutans complex based on BP&P and Bayes factor on the concatenated phylogenetic tree are highlighted by different colors as in Fig 1. The result of ABGD analyses for the mtSSU-single locus are shown in the picture. (TIF) [file pone.0216675.s001.tif]

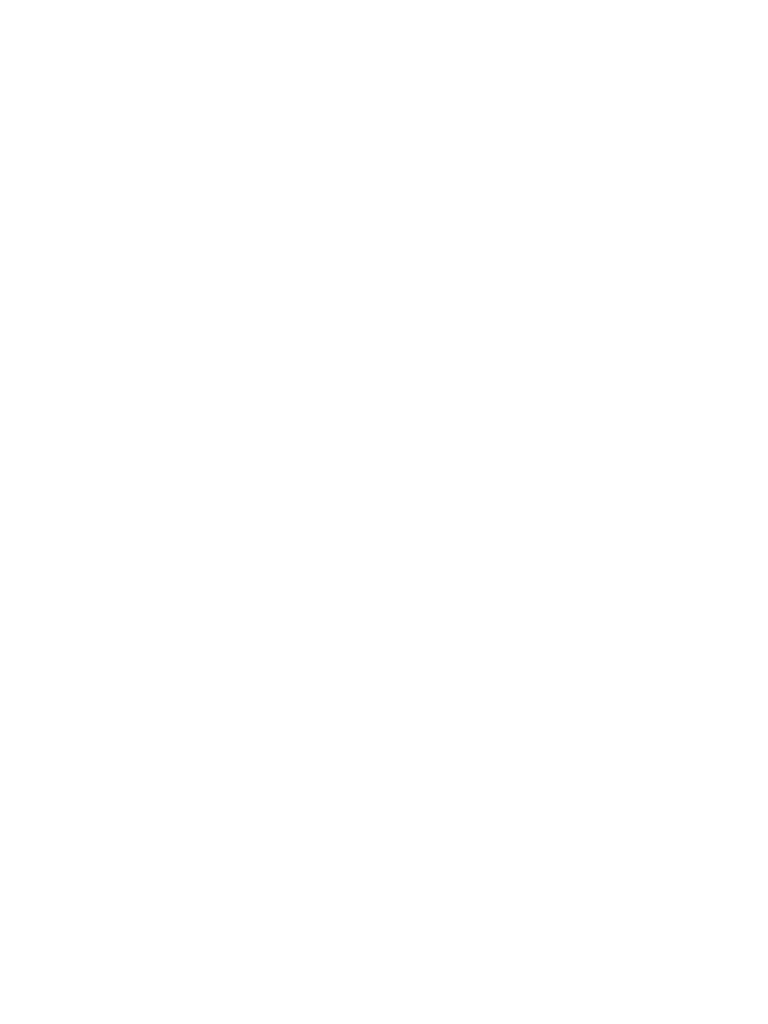

Supplement: S2 Fig — ML phylogenetic tree (IQ tree analysis) of Aspiciliella intermutans complex from MCM7 sequences. Bootstrap values are shown above their respective branches. Our six candidate species within the A. intermutans complex based on BP&P and Bayes factor on the concatenated phylogenetic tree are highlighted by different colors as in Fig 1. The result of ABGD analyses for the MCM7-single locus are shown in the picture. (TIF) [file pone.0216675.s002.tif]
